# The investigation of fluorescence and metal interaction properties of racemic 7,8,9,10-tetrahydro-3-hydroxy-4-(1-hydroxyethyl)benzo[c]chromen-6-one

**DOI:** 10.55730/1300-0527.3306

**Published:** 2021-08-18

**Authors:** Hayrettin Ozan GÜLCAN, Karar Tawfeeq SHUKUR, Açelya MAVİDENİZ, Okan SİRKECİOĞLU, Mustafa GAZİ

**Affiliations:** 1 Eastern Mediterranean University, Faculty of Pharmacy, Gazimağusa, TR. North Cyprus, Turkey; 2 Department of Chemistry, Faculty of Arts and Science, İstanbul Technical University, İstanbul, Turkey; 3 Department of Chemistry, Eastern Mediterranean University, Faculty of Arts and Science, Gazimağusa, TR. North Cyprus, Turkey

**Keywords:** Urolithin, synthesis, fluorescence, metal interaction

## Abstract

The design or investigation of fluorescence probes continues to receive attention with respect to the diverse applications of spectrofluorometry. Depending on the highly sensitive character, fluorescence spectroscopy-based methodologies have been widely used in recent years in different sciences, including analytical, environmental, and medicinal chemistry areas. In our previous works, we have shown the iron (III) selective on-off sensor properties of benzo[c]chromen-6-one derivatives. In this study, we have extrapolated this research to 4-substituted analogues and investigated both fluorescent and metal interaction properties. Following the synthesis and structure identification studies, (±)-7,8,9,10-tetrahydro-3-hydroxy-4-(1-hydroxyethyl)benzo[c]chromen-6-one was found as a fluorescent molecule displaying fluorescence enhancement in the presence of metals. This feature has been found quite different in comparison to the previous urolithins investigated. This finding suggested the substituent dependent effects and variations on the fluorescent properties of benzo[c]chromen-6-one system.

## 1. Introduction

The employment of fluorescence techniques and their applications have been increased worldwide in the last couple of decades with respect to the high sensitivity of this methodology [1–2]. In particular, the design of fluorescent molecules depending on the scientific requirements has led to the generation of diverse molecules with different scaffolds [3]. Diagnostic molecules, biomarkers, environmental pollutant sensitive agents are typical examples for the outcomes in the related scientific field [4–6]. Among them, the investigation of fluorescent changes depending on metals is another significant research area. On one hand, metal use increases globally with the development of technologies. On the other hand, it becomes critical to generate molecular sensing systems for both detection and quantification purposes [7–9].

The interaction of fluorescent molecules against metal ions can vary depending on the structural organization. Some fluorescent probes display Off-On characteristics. Mainly, a-non-fluorescent molecule gains fluorescent character with respect to its interaction with a specific metal or a group of metals [10,11]. There have been many off-on fluorescent probes designed and synthesized so far with diverse heterocyclic structures [11]. On the other hand, there are some fluorescent compounds lose their fluorescence property when it comes to interaction with another compound. This type of molecules has been referred to as On-Off probes [12,13]. There have also been many compounds introduced possessing this feature, particularly in the presence of metals. Overall, on-off, or off-on type fluorescence sensor applications are valuable tools for the diagnosis and quantification of metals.

Beside these, the metal interaction of specific fluorescence compounds can also trigger increase of the initial fluorescence intensity of a fluorescent probe. This process is referred to as fluorescence enhancement [14,15]. This type of application is not only utilized for the detection of metals but also for the development of more available systems in which the original fluorescent intensity is lower and needed to be upgraded for better fluorometric measurements [15–17].

In our previous studies, we have synthesized 3-hydroxy-6H-benzo[c]chromen-6-one (i.e. also referred to as Urolithin B) and discovered its on-off fluorescence probe property in the presence of iron (III) [18]. In the continuation of this work, the alternative hydroxyl substituted, methyl ether derivatives, and 7,8,9,10-tetrahydro-3-hydroxybenzo[c]chromen-6-one, the partial saturated form of Urolithin A, were synthesized, and it was observed that all these compounds, similar to Urolithin A, possess on-off fluorescent sensor character selective towards iron (III) [19–20]. All these alternative substitution patterns and the constant iron (III) sensor on-off character of the derivatives considered pointed out the significance of the lactone group in the interaction.

In order to question the effect of an electron withdrawing group on the fluorescence and metal interacting properties of these types of compounds, in this study, we have first aimed to synthesize 4-acetyl-7,8,9,10-tetrahydro-3-hydroxybenzo[c]chromen-6-one (i.e. THU-4-Ac), an acetylated derivative of partially saturated Urolithin B. On one hand, the presence of an electron-withdrawing group on the general fluorescence features has been aimed to be analyzed. On the other hand, the ketone function has been aimed to be reduced to obtain racemic 7,8,9,10-tetrahydro-3-hydroxy-4-(1-hydroxyethyl)benzo[c]chromen-6-one (i.e. THU-4-ALC), the alcohol derivative, to further see the changes in fluorescence properties, particularly in relation to metal interaction.

## 2. Experimental analysis

### 2.1. Materials and equipment

The chemicals and solutions employed in this study were reagent grade, and they were used upon obtaining without further purification. 2,6-Dihydroxyacetophenone, ethyl 2-oxocyclohexanecarboxylate, ZrCl_4_, NaBH_4_, ethanol, acetonitrile, MgCl_2_, PbCl_2_, KCl, NaCl, AgCl, BaSO_4_, ZnSO_4_.7H_2_O, Co(NO_3_)_2_.6H_2_O, Ni(NO_3_)_2_.6H_2_O, HgCl_2_, CuSO_4_, Fe(NO_3_)_3_.9H_2_O, and Al_2_(SO_4_)_3_ were all purchased from Sigma–Aldrich through the aid of local distributors in Turkish Republic of Northern Cyprus. The solutions of metal ions were prepared in ultra-pure distilled water.

A thermoscientific spectrofluorometer (Varioskan Flash model multi-plate reader) was employed for the fluorescence measurements. Thin-layer chromatography studies were conducted on Merck aluminum-packed silica gel plates employing ethyl acetate / n-hexane as mobile phase at 1:1 and 1:3 ratios. An Electrothermal IA 9200 Model melting point apparatus was used to measure the melting points, and the data presented were uncorrected. Elemental analysis was performed using a Flash Smart model ThermoFisher elemental analyzer. In order to obtain the infrared spectrums of the title molecules, FTIR Prestige 21 model Shimadzu instrument was employed. An Agilent VNMSR-500 NMR spectrometer was used to get the NMR spectrums of the title molecules. CDCl_3_ was used as solvent with the presence of tetramethylsilane as internal standard, and the chemical shifts were presented in ppm (δ).

### 2.2. Synthesis of 3-hydroxy-7,8,9,10-tetrahydro-6H-benzo[c]chromen-6-one (THU-OH)

THU-OH was synthesized according to the previously published methodology [20–21]. Briefly, 0.22 g resorcinol, 0.37 g ethyl 2-oxo-cyclohexancarboxylate, and 0.4 g ZrCl_4_ were mixed in a 20 mL reaction flask and heated at 85 °C for 1 h. The precipitate formed was filtered off and washed with ice-cold water. The spectral analysis of the compound was found identical with our previous findings [20–21].

### 2.3. Synthesis of (±)-4-acetyl-7,8,9,10-tetrahydro-3-hydroxybenzo[c]chromen-6-one (THU-4-Ac)

6.5 mmol acetophenone, 6.8 mmol of ethyl 2-oxocyclohexanecarboxylate, and 2.5 mmol of ZrCl_4_ was heated at 95 °C for 1 h under neat conditions. At the end of the period, 10 mL of ice-cold water was added, and the precipitate formed was filtered off. Light yellow powder. Melting point 143°C (uncorrected data). IR: 1669 cm^−1^. ^1^H NMR (500 MHz, Chloroform-d) δ 13.43 (s, 1H), 7.63 (d, J = 9.0 Hz, 1H), 6.87 (d, J = 8.9 Hz, 1H), 2.95 (s, 3H), 2.73 (q, J = 5.8 Hz, 2H), 2.55 (t, J = 6.3 Hz, 2H), 1.95 – 1.70 (m, 4H). ^13^C NMR (125 MHz, Chloroform-d) δ (ppm): 204.1, 163.8, 161.5, 150.2, 149.1, 133.7, 122.2, 118.5, 113.4, 111.9, 30.8, 28.7, 24.8, 24.3, 23.7. Yield obtained 88%. Anal. calc. for C15H14O4: C 69.76, H 5.46; found C 69.82, H 5.44.

### 2.4. Synthesis of (±)-7,8,9,10-tetrahydro-3-hydroxy-4-(1-hydroxyethyl)benzo[c]chromen-6-one (THU-4-ALC)

A total of 13.2 mmol of sodium borohydride is dissolved in 10 mL ethanol and added dropwise to the solution of 5.5 mmol of THU-4-Ac in 10 mL ethanol. Following stirring at room temperature for 20 minutes, the mixture was acidified with 20 mL of 1N HCl and extracted with ethyl acetate (3 × 50 mL). The organic layer collected was added dry magnesium sulfate, and it was filtered off. The product was obtained upon the evaporation of the organic phase under reduced pressure. White-yellow powder. Melting point 121 °C (uncorrected data). IR: 1628 cm^−1^. ^1^H NMR (500 MHz, Chloroform-d) δ 9.75 (s, 1H), 7.31 (d, J = 8.8 Hz, 1H), 6.81 (d, J = 8.7 Hz, 1H), 5.88 (q, J = 6.6 Hz, 1H), 4.62 (bs, 1H), 2.69 (q, J = 5.8 Hz, 2H), 2.47 (t, J = 6.3 Hz, 2H), 2.08 – 1.70 (m, 7H). ^13^C NMR (125 MHz, Chloroform-d) δ 162.5, 151.8, 150.7, 147.5, 127.4, 122.1, 118.6, 112.5, 111.4, 58.7, 31.1, 24.8, 23.6, 23.0, 22.9. Yield obtained 81%. Anal. calc. for C15H16O4: C 69.22, H 6.20; found C 68.87, H 6.49.

### 2.5. Fluorescence studies

Employing a Varioskan Flash model multi-plate reader spectrofluorometer, the fluorometric measurements were performed. A total of 1 mM solutions of the title molecules (i.e. THU-OH, THU-4-Ac, and THU-4-ALC) were prepared in Acetonitrile – Water (9:1), and they were screened to measure the excitation λmax. The emission spectrums were obtained accordingly for the compounds display fluorometric properties. The aqueous solutions of selected metal ions were prepared in appropriate stock solutions, and the effect of metal solutions on fluorescence intensity of fluorescence title molecules were investigated in 1:1 probe-metal ratio.

## 3. Results and Discussion

**Figure 1:**
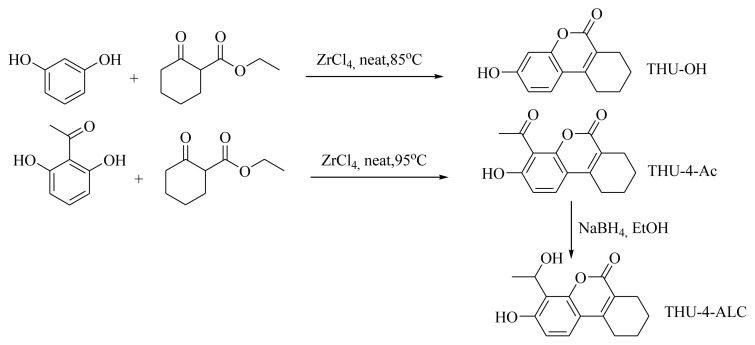
The synthetic scheme of the title molecules.

The synthetic scheme is provided in Figure 1 for the title compounds. THU-OH was obtained according to the protocol previously published [20]. The 4-acetyl and 4-(1-hydroxyethy) derivatives of THU-OH were obtained in good yields. The spectral and the elemental analysis of the compounds was employed for the structure identification studies.

The fluorescence scanning of THU-4-OH was found identical with our previous measurements [20]. The UV spectrum of THU-4-Ac has revealed out 320 nm as the wavelength for the maximum absorption. The emission spectrum based on this λmax generated no emission. In other words, THU-4-Ac did not display any fluorescent property. This observation was critical, since we have shown that benzo[c]chromen-6-one derivatives with hydroxyl-, or methoxy-substituents possess fluorescent characteristics and an electron-withdrawing group, such as in the case of 4-acetyl substituted derivative in THU-4-Ac have blocked this feature.

**Figure 2:**
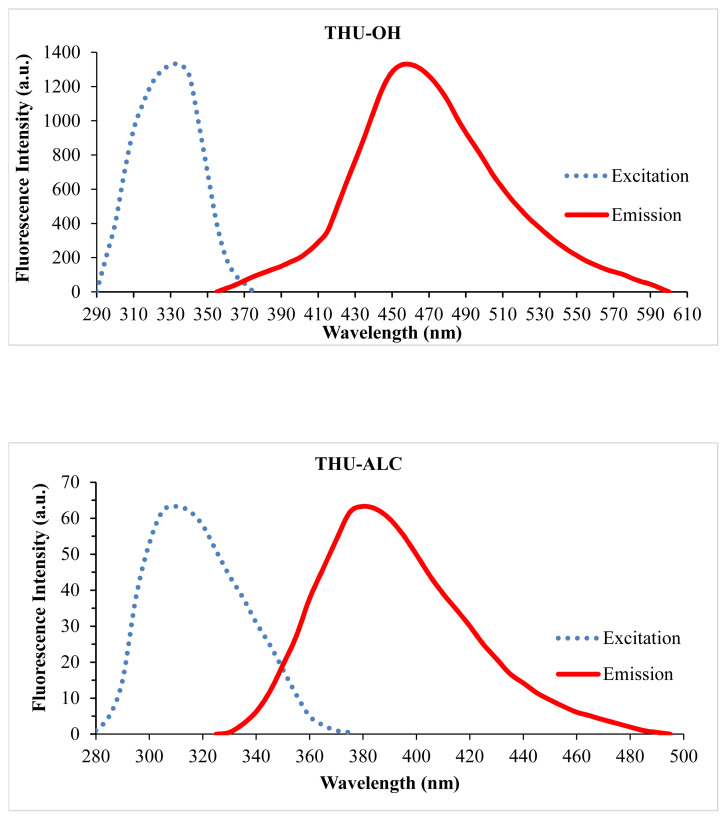
The excitation and emission spectra of THU-OH and THU-ALC.

Employing a reduction reaction on THU-4-Ac yielded out its corresponding secondary alcohol racemic molecule, THU-4-ALC. The UV spectrum generated a λmax at 310 nm. Therefore, the presence of the 1-hydroxyethyl substituent on the 4th position generated a hypsochromic shift in comparison to its 4-deacetylated analogue, THU-OH. Further scanning for the emission spectrum revealed out the fluorescent characteristics of THU-4-ALC. Therefore, the fluorescence property lost was gained back with the reduction of ketone function. In other words, the removal of the electron withdrawing character of the substituent on the 4th position has made the benzo[c]chomen-6-one scaffold retain its fluorescent properties. The excitation and emission spectrum characteristics of the title molecules are shown in Figure 2.

In our previous studies, we have pointed out the specific interaction of urolithins and a partially saturated analogue, THU-OH, with iron (III). The presence of iron (III) resulted in the quenching of fluorescent properties of these compounds. Mainly, urolithins were shown to act as On-Off probes in the presence of iron (III). Since the title molecule, THU-4-Ac, was found a non-fluorescent molecule, we first examined whether the compound can act as off-on probe in the presence of different metals. It was observed that the 4-acetyl derivative of THU-OH did not display any fluorescent feature in the presence or absence of any metals.

**Figure 3:**
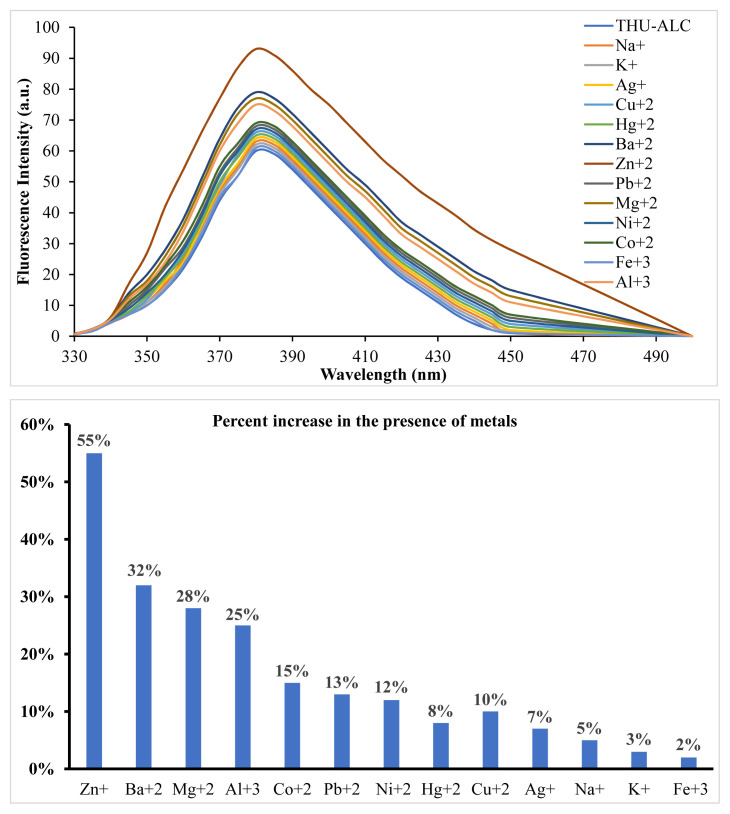
Fluorescence enhancement of THU-ALC through interaction with selected metals.

The reduced form of THU-4-Ac, the THU-4-ALC compound, was shown to have fluorescent properties. As seen in Figure 2, the fluorescence intensity of the molecule appeared to be lower in comparison to the fluorescence intensity of THU-OH at the same concentration. The interaction of THU-4-ALC with metals was also investigated with fluorescence titrations. The initial experiments with the selected metals have shown that almost all of the metals employed resulted in the enhancement of fluorescence intensity at varying degrees. Particularly, Zn^+^, followed by Ba^+2^, Mg^+2^, and Al^+3^, generated an apparent increase in the fluorescence intensity of THU-4-ALC. This observation was totally different from the fluorescent characteristics of THU-4-OH. The results obtained are shown in Figure 3.

**Figure 4:**
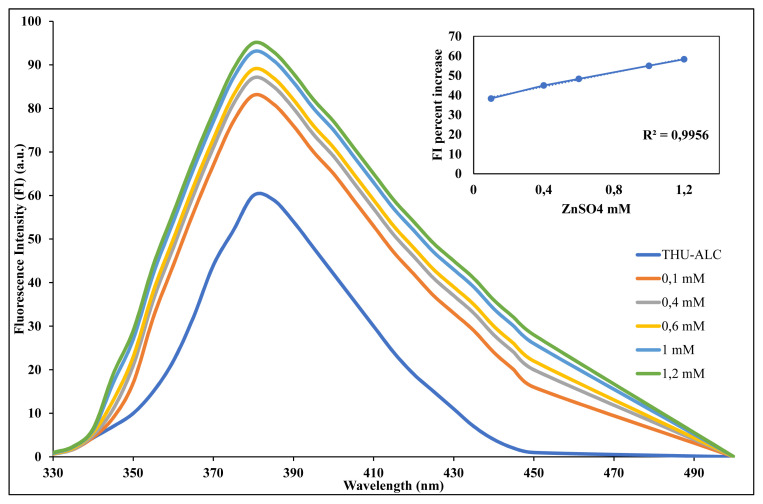
Fluorescence enhancement with increasing concentration of Zinc.

In the continuation of this study, another set of experiments was planned to measure the concentration-dependent effect on the metal-induced enhancement of fluorescence intensity. With respect to the highest effect obtained with zinc, its different concentrations were prepared and titrated with the constant concentration of the fluorescence probe (i.e. THU-4-ACL). As seen in Figure 4, the effect was observed concentration-dependent.

The overall results have pointed out that the fluorescence properties of benzo[c]chromen-6-one bearing systems are substituent-dependent. Particularly, electron-withdrawing groups can terminate the fluorescence property, whereas the on-off character might be also replaced with fluorescence enhancement properties in the presence of alternative substituents.

## 4. Conclusion

Within the concept of this research, two alternative derivatives of a previously shown fluorescent molecule, THU-OH, were prepared and screened both for their fluorescence properties and the change in fluorescence in the presence of different metals. Different from THU-OH, an iron (III) selective on-off probe, the title molecule THU-4-Ac was found non-fluorescent. Therefore, it was shown apparently that electron-withdrawing groups can totally convert the system to a non-fluorescent molecule. Moreover, this compound did not display any off-on character in the presence of selected metals. The reduction of THU-4-Ac yielded out the THU-4-ALC molecule, and the fluorescence measurements on this molecule have shown its fluorescence characteristics. Although fluorescence intensity was lower in comparison to the fluorescence of THU-OH, the compound displayed quite distinct features in terms of interaction with metals. Unlike the iron (III) selective On-Off probe character of THU-OH, the fluorescent feature of THU-4-ALC was not affected too much with iron (III). More important than that THU-4-ALC displayed fluorescence enhancement in the presence of majority of the metals employed. The highest effect was observed with zinc. Besides, the effect of zinc was found concentration dependent.

**Figure 5:**
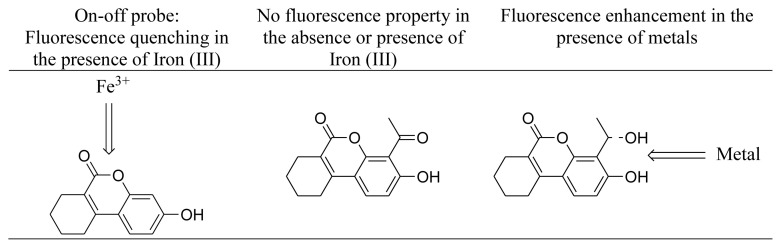
Different interaction of the probes with iron (III).

The difference observed between THU-OH and THU-4-ALC might be related to the different interaction characteristics of these compounds with different metals. At first hand, it might be postulated that the lactone group in THU-OH is not sterically hindered in comparison to THU-4-ALC. On the other hand, the additional alcohol functional group within the vicinity of the phenolic hydroxyl on THU-4-ALC can generate an energetically and sterically optimum pocket for the interaction of diverse metals. The net fluorescence enhancement character obtained might be mechanistically explained via this approach (Figure 5). However, these results warrant not only the design of different benzo[c]chromen-6-one derivatives with alternative substitutions to see the fluorescence properties and its change in the presence of different metals but also additional complex crystallization studies to ensure the mode of interaction between these probes and metal ions.
